# A case of paradoxical vocal cord movement misdiagnosed as anaphylaxis 

**DOI:** 10.5414/ALX02502E

**Published:** 2024-07-04

**Authors:** Mustafa Ilker Inan, Yasemin Akgul Balaban, Sait Yesillik, Ozgur Kartal

**Affiliations:** Ankara Gulhane Training and Research Hospital, Division of Immunology and Allergic Diseases, Ankara, Turkiye

**Keywords:** paradoxical vocal cord movement, anaphylaxis

## Abstract

Introduction: Anaphylaxis is a severe and life-threatening systemic hypersensitivity reaction. The most frequently encountered causes are foods, drugs, and bee venom, but anaphylaxis may also occur idiopathically. Paradoxical vocal cord movement (PVCM), is a cause of upper airway obstruction due to abnormal adduction of vocal cords during inspiration and, to some degree on expiration. It may be misdiagnosed as asthma or anaphylaxis, and there may be delays in diagnosis. Case report: We present a 20-year-old male patient with coexistence of urticaria and stridor findings who was evaluated and treated as having idiopathic anaphylaxis but then was diagnosed with PVCM after recurrence of stridor attacks. Conclusion: It is useful to bear the diagnosis of PVCM in mind in patients with recurrent and unexplained stridor or in patients with stridor that does not improve despite treatment for another diagnosis such as anaphylaxis. This way, administration of epinephrine, high-dose corticosteroids and interventions such as intubation or tracheostomy can be avoided.

## Introduction 

Anaphylaxis is a severe and life-threatening systemic hypersensitivity reaction that suddenly occurs depending on the mast cell and basophil-derived mediators after exposure to an allergen [1]. Even though the most frequently encountered causes of identifiable anaphylaxis are foods, drugs, and bee venom, anaphylaxis may also occur idiopathically and the triggering factor may not be detected [2, 3]. 

Paradoxical vocal cord movement (PVCM), the etiology of which is not well understood, is a cause of upper airway obstruction due to abnormal adduction of the vocal cords during the inspiration and, to some degree on expiration [4, 5]. It mostly appears with severe acute respiratory distress and stridor findings, may be misdiagnosed as asthma or anaphylaxis, and there may be delays in diagnosis [4]. 

In this article, we present a case that occurred with the coexistence of urticaria and stridor findings which was evaluated and treated as idiopathic anaphylaxis, but then was diagnosed as PVCM after the recurrence of stridor attacks. 

## Case report 

A 20-year-old male patient, diagnosed with chronic idiopathic urticaria ~ 2 years previously and whose urticaria was under control with a regular treatment of cetirizine 20 mg/day, complained of itching and blisters on his body and a sudden shortness of breath at ~ 11:00 AM the day before his presentation to our outpatient clinic. The patient pointed out that he did not take any medications and had not consumed any food and beverages until 3 hours before the onset of his complaints, and did not do exercise 3 hours before the onset of his complaints. Due to the patient’s current complaints, pheniramine (45.5 mg IM) and dexamethasone (8 mg IM) were administered by the on-site physician at the patient’s workplace, and his rashes resolved, but his shortness of breath did not subside. Therefore, he was transferred to emergency service of a secondary healthcare hospital by an ambulance. He received pheniramine (45.5 mg IV) and methylprednisolone (80 mg IV). He was kept under observation for 5 hours, and when his shortness of breath had partially subsided, he was discharged. 

As his shortness of breath worsened again at home, the patient presented to a tertiary hospital emergency department at ~ 07:00 PM. During his examination in the emergency service, his vital signs were found to be normal, but uvular edema was observed and stridor was heard. The patient had a pre-diagnosis of anaphylaxis and received adrenaline (0.5 mg IM), pheniramine (45.5 mg IV) and methylprednisolone (250 mg IV), salbutamol (2.5 mg nebule), and budesonide (1 mg nebule). After these treatments, the patient’s respiratory complaints improved during the observation in the emergency service. However, his shortness of breath worsened again in the morning at ~ 04:00 AM. Due to ongoing stridor of the patient, thorax CT scan was taken and it was found to be normal. Ear-nose-throat (ENT) consultation was requested, where both vocal cords were observed to be mobile, and no mass or mucosal irregularity was observed in the vocal cords, and no upper airway obstruction that would cause stridor was found. Upon pulmonologist consultation, no pathology was found to cause lower airway obstruction. 

On his physical examination in our outpatient clinic, SpO_2_ was 99%, blood rate was 105/min, blood pressure was 140/80 mmHg, no uvular edema and urticaria/angioedema were observed. Inspiratory/expiratory stridor was heard. When the history of the patient was evaluated, also considering the late administration of the first dose of adrenaline, a diagnosis of prolonged anaphylaxis with a skin and respiratory system involvement was suspected. Adrenaline (0.3 mg IM) was administered to the patient. Due to inability to provide complete relief in respiratory complaints and physical examination findings, the same dose of adrenaline was administered 15 minutes later. After this administration, his stridor significantly relieved. 

He was hospitalized for follow-up. Approximately 6 hours later, his shortness of breath worsened again, and stridor, similar to that during the physical examination in the outpatient clinic, developed without accompanying uvular edema. He was administered 2 mg of nebulized adrenaline, and his stridor relieved again after the treatment. However, due to recurrent stridor attacks during his hospital stay, nebulizer treatment (alternately adrenaline, salbutamol/budesonide) was given to him every 6 hours. SpO_2_ was found to be normal in all of the stridor attacks of the patient. 

**Figure 1:**
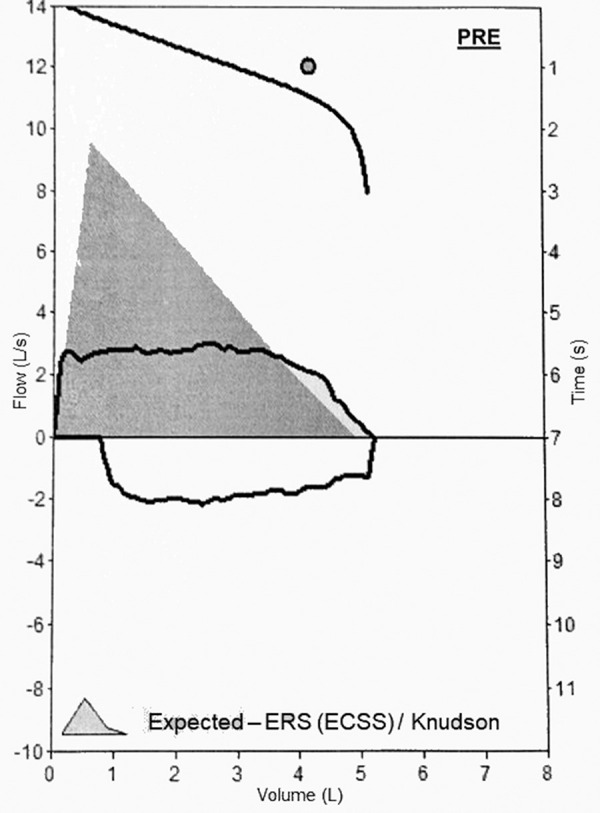
Flattening in both inspiratory and expiratory flow volume curves in pulmonary function test.

In the laboratory tests, hemogram, routine biochemistry, and CRP values were normal. Serum tryptase level obtained 24 hours after his presentation to the emergency service was normal (2.31 μg/L, normal value < 11.4 μg/L). C1 esterase inhibitor (36.5 mg/dL, normal value 18 – 40 mg/dL) and C1 esterase inhibitor function (110%, normal value 70 – 130%) results were normal. CT of the neck was found to be normal. Pulmonary function test (PFT) was performed, and a flattening compatible with fixed airway obstruction was observed in both inspiratory and expiratory flow volume curves (Figure 1). Therefore, a paradoxical movement was observed in the bilateral vocal cords during the endolaryngeal endoscopic examination of the patient who was re-evaluated by the ENT. Respiratory physiotherapy was planned for the patient whose opening of rima glottidis was observed to be adequate and who was not observed to have vocal cord paralysis or external pathology in other laryngeal structures. 

Six weeks after the patient’s discharge, skin prick tests were performed with inhalant allergens and the most common food allergens in adults including cow’s milk, sheep’s milk, wheat, egg white, egg yolk, red meat, white meat, soy, peanut, hazelnut, walnut, almond, and fish, and all of the results were found to be negative. 

Gastroenterology, neurology, and psychiatry consultations were performed to exclude the differential diagnoses (gastroesophageal reflux disease (GERD), neurological and psychogenic conditions), and no pathology was found. His PVCM was relieved by respiratory physiotherapy. 

## Discussion 

Although the incidence is not exactly known, it has been reported that PVCM diagnosis affects 2.5 – 22% of patients who frequently apply to emergency services with a sudden onset of dyspnea [6]. It has been reported that psychogenic disorders, neurological diseases, GERD, inflammation, viral upper airway diseases, and extubation after anesthesia may have a role in its etiology [4, 5, 7]. 

During the treatment for PVCM, patient education with speech therapy as well as respiratory physiotherapy with effective breathing techniques such as pursed lip breathing, breathing through a narrow straw, and panting are important [4, 5, 8]. 

The tests and examinations found no organic pathology in our case, and the current PVCM was considered to be due to a psychogenic disorder. The patient’s symptoms were relieved with psychiatric advices and respiratory physiotherapy. 

During normal breathing, the vocal cords are open, and they only close during swallowing, coughing, speaking, and holding breath. Although the PVCM mostly occurs in the inspiratory phase of breathing, did not do exercise 3 hours before the onset of his complaints [4, 5, 8, 9, 10]. This condition explains the flattening in the flow volume curves observed in both the inspiratory and expiratory phases of our patient’s PFT. 

In the literature, there are few cases in which PVCM was found together with stridor during inspiration and expiration. Jérôme et al. [11] reported a patient with post-COVID-19 condition who had PVCM that occurred during both inspiration and expiration while speaking. Bahrainwala et al. [12], on the other hand, reported a patient with vocal cord adduction during both inspiration and expiration in laryngoscopy, but only flattening in the expiratory flow volume curve in PFT. 

PVCM may be triggered by exercise, perfumes, odors, and emotional stress [4, 5, 7]. The reason why no pathology could be found in our patient despite the ENT evaluation and direct laryngoscopic examination performed twice in the emergency service, can be explained by the thought that laryngoscopy could be normal when there is no triggering factor. Due to the symptom relief the patient experienced from the treatments he received before the ENT evaluation, his emotional state, which we think is the current triggering factor, may have improved. 

Direct visualization of the vocal cords by laryngoscopy during an attack is the gold standard in the diagnosis PVCM [4, 5, 6, 9]. Since it is difficult to perform this procedure during an attack, both in terms of patient compliance and performing the procedure immediately, there may be delays in diagnosis. It has been reported that the average duration from symptom onset to diagnosis is more than 4 years [13]. Therefore, it is useful to bear the diagnosis of PVCM in mind in patients with recurrent and unexplained stridor or in patients with stridor that does not improve despite treatments for another diagnosis such as asthma or anaphylaxis. 

**Figure 2:**
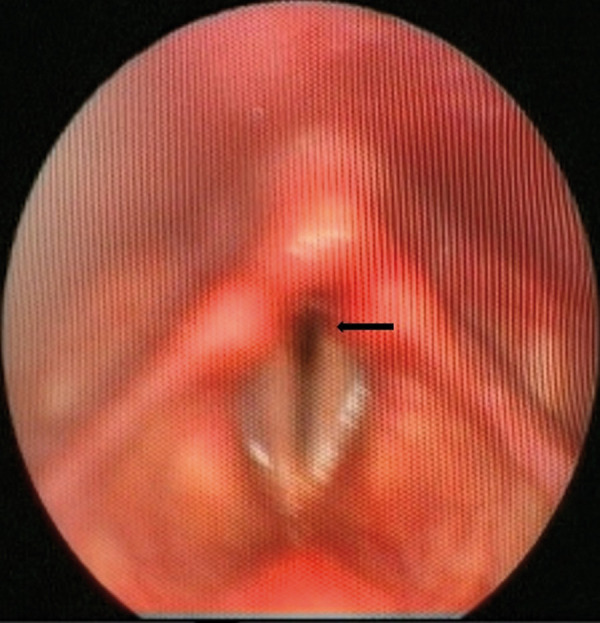
Abnormal adduction of the vocal cords during inspiration of the patient. The black arrow shows the posterior glottal chink.

A not immediately understood and sometimes identifiable finding during the PVCM, but stated to be pathognomonic for PVCM, is a small opening occurring in the posterior glottis during inspiration and called posterior glottal chink [4, 9]. In our patient, a posterior glottal chink finding was observed during laryngoscopy examination performed for the third time during his attack (Figure 2). 

Anaphylaxis can be diagnosed with the presence of at least one of the symptoms associated with the skin and mucosal findings such as generalized urticaria, itching, and rash as well as swelling of the lips/tongue/uvula that occur acutely (within minutes to hours), together with respiratory system findings such as dyspnea, wheezing, bronchospasm, stridor, hypoxemia, or end organ dysfunction such as decrease in blood pressure, hypotonia (collapse), syncope, incontinence [14]. 

The main reasons that made us think of a diagnosis of prolonged anaphylaxis were the absence of stridor in our patient’s previous history, the simultaneous onset of urticaria and stridor, detection of uvula edema in the examination by emergency service, the presence of tachycardia, and the late administration of adrenaline injection to the patient. In particular, the fact that stridor occurred for the first time simultaneously with urticaria suggests that anaphylaxis itself may cause PVCM. 

Garcia-Neuer et al. [8] reported a case of drug-related anaphylaxis who received epinephrine treatments multiple times; however, they pointed out that the diagnoses that had led to previous epinephrine use could have been wrong, since the patient’s history of requiring epinephrine did not recur after being diagnosed with PVCM. In a retrospective study conducted by O’Connell et al. [15], it was reported that 2 of the 20 patients diagnosed with PVCM were previously misdiagnosed as anaphylaxis, and 15 were misdiagnosed as asthma. 

If these patients cannot be correctly diagnosed, their conditions may be confused with, e.g., anaphylaxis or asthma, and may patients be exposed to unnecessary treatments. Furthermore, they could be exposed to serious conditions such as repeated intubation due to acute attacks of respiratory failure [15, 16]. 

In conclusion, in addition to idiopathic anaphylaxis, PVCM should also be kept in mind among the differential diagnoses in order to prevent administration of epinephrine, high-dose corticosteroids, and interventions such as intubation or tracheostomy in patients who present to the emergency service with a sudden onset of stridor. The ENT physician should also be warned about this issue, and as we did in this case, attention of the ENT physician should be paid to vocal cord movements in laryngoscopic examination. 

## Authors’ contributions 

Inan MI: evaluation and follow-up of the patient; writing, drafting, revising of the manuscript. Balaban YA: follow-up of the patient; drafting, revising of the manuscript. Yesilik S: evaluation and follow-up of the patient; revising of the manuscript. Kartal O: follow-up of the patient; revising of the manuscript. 

## Funding 

None. 

## Conflict of interest 

None.
